# Development and Validation of an Openable Spherical Target System for High-Precision Registration and Georeferencing of Terrestrial Laser Scanning Point Clouds

**DOI:** 10.3390/s25247512

**Published:** 2025-12-10

**Authors:** Maria Makuch, Pelagia Gawronek

**Affiliations:** Department of Land Surveying, University of Agriculture in Krakow, 21 Mickiewicza Ave., 31-120 Krakow, Poland; pelagia.gawronek@urk.edu.pl

**Keywords:** reference targets, point cloud registration, georeferencing accuracy, 3D technology measurement, engineering geodesy, multi-sensor data integration

## Abstract

Terrestrial laser scanning (TLS) point clouds require high-precision registration and georeferencing to be used effectively. Only then can data from multiple stations be integrated and transformed from the instrument’s local coordinate system into a common, stable reference frame that ensures temporal consistency for further analyses of displacement and deformation. The article demonstrates the validation of an innovative referencing system devised to improve the reliability and accuracy of registering and georeferencing TLS point clouds. The primary component of the system is openable reference spheres, whose centroids can be directly and precisely determined using surveying methods. It also includes dedicated adapters: tripods and adjustable F-clamps with which the spheres can be securely mounted on various structural components, facilitating the optimal distribution of the reference markers. Laboratory tests with four modern laser scanners (Z+F Imager 5010C, Riegl VZ-400, Leica ScanStation P40, and Trimble TX8) revealed sub-millimetre accuracy of sphere fit and form errors, along with the sphere distance error within the acceptance threshold. This confirms that there are no significant systematic errors and that the system is fully compatible with various TLS technologies. The registration and georeferencing quality parameters demonstrate the system’s stability and repeatability. They were additionally verified with independent control points and geodetic levelling of the centres of the spheres. The system overcomes the critical limitations of traditional reference spheres because their centres can be measured directly using surveying methods. This facilitates registration and georeferencing accuracy on par with, or even better than, that of commercial targets. The proposed system serves as a stable and repeatable reference frame suitable for high-precision engineering applications, deformation monitoring, and longitudinal analyses.

## 1. Introduction

Terrestrial Laser Scanning (TLS) has become the central surveying technology for the remote, fully automated acquisition of spatial coordinates for millions of points in near real time [1]. Its potential lies in remote, quick, and effective collection of reliable spatial data in high resolution and with sub-millimetre accuracy. These advantages have driven its expansion into multiple domains, including high-precision engineering surveys [2,3,4,5]. Terrestrial laser scanning is especially important and effective in the practice and research of deformation monitoring, where high accuracy and longitudinal consistency are equally vital [6,7,8]. The numerous case study reports, which often involve verification against traditional survey methods, confirm the value of TLS data in displacement and deformation analyses of strategic engineering structures, such as hydraulic infrastructure [9,10,11,12,13], underground facilities [14,15,16,17], bridge structures [18,19,20,21,22,23], or towers [24,25,26,27,28]. Still, TLS deformation monitoring would not be possible without precise registration and georeferencing of cloud points. These steps are necessary to integrate scans from various stations and to transform data from the instrument’s local coordinate system into a stable reference frame that ensures longitudinal consistency for deformation analyses [6,8,29]. Authors have emphasised the urgency of highly accurate TLS data registration and georeferencing in deformation monitoring time and time again [29,30,31]. Yang et al. [32] position registration and georeferencing as the fundamental activities for comparing multi-epoch scans. Their high quality is counted among the primary challenges of TLS research on dynamic construction sites. Shen et al. [8] argue that registration precision is meaningful for further analyses, especially regarding the avoidance of systematic errors when estimating deformation parameters. As Mukupa et al. [3] highlighted, the transformation of registered TLS point clouds into a higher-level reference frame is central for post-processing in deformation monitoring. Lague et al. [33] believe orientation uncertainty to be a main source of errors in longitudinal analyses of point clouds. Lindenbergh and Pietrzyk corroborate this [34], stating that registration and georeferencing significantly affect the overall error budget and that their contributions should be minimised. Even the slightest discrepancies in point cloud transformation can mimic actual displacements and deformations, leading to unwanted measurement noise and significantly affecting interpretative reliability [6]. Therefore, TLS data orientation is critical for deformation monitoring [24], and the dominance of registration and georeferencing errors should be curbed [35].

Recent decades saw a dynamic growth in the palette of TLS data registration techniques: from methods involving manual intervention, fully automated coarse-to-fine algorithms (initial approximation of translation and rotation parameters followed by precise registration), to deep learning-based registration [30,31,36,37,38,39,40]. Despite the abundance of novel proposals, the traditional method based on artificial reference markers (target-based method) remains the most reliable and most popular among engineers [4,7,29,41,42,43,44]. This method, also known as the indirect method, is most often employed not only for registering but also for georeferencing TLS data. It ensures reliable estimation of transformation parameters and stitching of all point clouds for the object in a coherent reference frame [3,8,45]. The target-based method is more accurate than direct methods that determine the instrument’s position and orientation to ‘directly’ acquire the point cloud in the reference frame [3,46,47,48]. The foundation of registering and georeferencing point clouds with the target-based method is to use properly distributed and securely mounted reference markers within the scanner’s field of view, such as white spheres or high-contrast flat targets [41,49,50]. These objects are easily identifiable on scans thanks to their specific colour or shape. They can be detected automatically to spare human intervention and speed up orientation [51,52,53]. This method is independent of the scanned object, conveniently yields the desired geometric layout, and gives easily interpretable results [43]. Although the target-based method is mature [15], researchers continue to improve existing reference markers or propose new ones tailored to specific applications [50,53,54,55,56]. A new target type proposed by Janßen et al. [54] is an improved version of a standard black-and-white target with an eight-fold radial design. These authors demonstrated that the target reduced uncertainty in target centre identification compared with conventional, commercial black-and-white targets. Singh et al. [53] designed unique 3DUID markers: flat, square panels with a black-and-white gradient and a triangular notch in the upper part for direct surveying. The idea behind the design was to address issues of georeferencing and registering point clouds under difficult conditions, like in underground mines. A new, cheap design of a high-contrast target made from a CD-ROM was proposed by Lichti et al. [50]. The authors also wrote an algorithm for finding the target’s centre dedicated to registering point clouds and calibrating scanning systems. Still, recent inventions go beyond flat markers. Muralikrishnan et al. [57] introduced plate-sphere targets. These hybrids consist of a square plate with a centrally positioned sphere. Their design combines the benefits of flat and spherical targets, facilitating more accurate assessment of ranging errors of laser scanners.

However, reference markers may introduce systematic errors to the observations. These have to be identified and eliminated in high-precision applications [58]. Targets’ Geometry Distribution (TGD) is critical for correct registration, georeferencing, and target acquisition accuracy [3,41,44]. The registration error is inversely proportional to the number of targets and the total distance between them and their centre of mass, as Fan et al. [29] demonstrated. Bornaz et al. [59] concluded that the accuracy of registration of two scans heavily depends on the data overlap ratio (the configuration of targets in the scan overlap area). They set the minimum overlap at 30% to achieve accuracy comparable with the instrument’s ranging precision. On the other hand, Yang et al. [60] found that the accuracy of the target-based method hinges primarily on the precision of extraction of the tie point, which depends on the scanning incidence angle for all shapes except for a sphere. Any displacement of reference markers also adds to orientation errors. Manual rotation of the targets towards the scanner increases time consumption and may introduce unintended displacement, which disqualifies the target [41,58]. In this regard, spherical targets have a significant advantage over flat markers because their geometry facilitates unambiguous determination of their centre (tie point) without reorienting [52,61]. Becerik-Gerber et al. [41] demonstrated that reference spheres may improve registration accuracy by 42–65% compared to traditional—flat or paddle—targets, whose performance is limited by the incidence angle [48,54,62]. The latest research by Muralikrishnan et al. [63] confirmed systematic, angle-dependent sub-millimetre errors of target centres, which affect registration accuracy directly. No such errors were found for reference spheres. The good registration performance of spheres has been confirmed by Gümüş et al. [64], who demonstrated their statistically better results compared to methods using specific points and surface matching. The literature also emphasises the role of sophisticated algorithms for automated sphere detection and sphere centre extraction, techniques that significantly improve the performance and accuracy of registration procedures [60,61,65,66,67]. New algorithms, such as PK-RANSAC, improve the precision and robustness of sphere centre extraction, even in noisy conditions or for partially occluded objects [67]. According to Yang et al. [60], isotropic characteristics of spheres make them the go-to system for target-based point cloud registration. Moreover, Shi et al. [66] emphasised that spherical targets are important for registration as well as verification and calibration of instruments and measurement accuracy assessment [57,68,69,70]. Still, despite the critical advantage of reference spheres, which is their omnidirectionality, obviating the need to reorient them, their primary downside is poor georeferencing performance, as made clear by Fryskowska [71]. This is because the coordinates of the centroid of standard spheres cannot be measured directly, which complicates their unambiguous integration into the reference frame.

- This article proposes an innovative referencing system to improve the reliability of TLS data registration and georeferencing. The system comprises:
- reference spheres whose centroids can be determined precisely with surveying methods,
- dedicated adapters for securing the spheres to diverse objects (such as flat surfaces, railings, and balustrades) for the optimal placement of the targets.
- The system facilitates a precise stitching of point clouds acquired from multiple stations, transformation of TLS data to a common coordinate system, and their integration with data obtained using traditional surveying methods (tacheometry, geodetic levelling, GNSS). The system’s performance was verified under laboratory conditions using four terrestrial laser scanners from leading brands.

## 2. Materials and Methods

### 2.1. Novel Openable Spherical Target System

The central part of the proposed target system for TLS data registration and georeferencing is an openable reference sphere designed in line with the latest literature recommendations for new targets [54,56]. The sphere’s steel is both mechanically strong and has a low coefficient of thermal expansion, ensuring dimensional stability under variable conditions. The sphere’s diameter is 150 mm, which the literature recommends as the optimal value for TLS point cloud registration [72]. It is a hollow ball made up of two precisely matching hemispheres. They are assembled with three spring-loaded catches positioned at a fixed interval along the sphere’s circumference to ensure sphericity and stability of the centroid after assembly. There is a threaded bush at the bottom of the reference sphere for stabilising and adjusting the height of a cylindrical centring pin. The pin has a strictly defined centre at the end, compatible with a high-precision spherical prism (retroreflector) to ensure coaxial positioning of the hemispheres. Thanks to the adjustable pin height, it can be used as a marker in traditional surveying, such as tacheometry, geodetic levelling, or even GNSS observations, which substantially improves the system’s functionality. As recommended in [73,74,75], the outer surface of the sphere has appropriate reflection characteristics. Its white, smooth and matte coating has a high albedo, which minimises noise and helps with automated target detection in point clouds.

**Figure 1:**
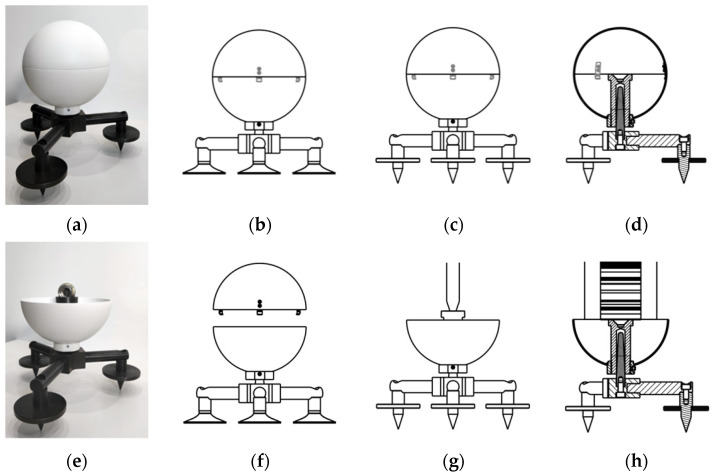
Tripod adapter with an openable reference sphere: (**a**) perspective with a closed sphere; (**b**) side view with suction cups and a closed sphere; (**c**) side view with fang stabilisers and a closed sphere; (**d**) cross-section view with fang stabilisers and a closed sphere; (**e**) perspective with an open sphere and a retro-reflector; (**f**) side view with suction cups and an open sphere; (**g**) side view with an open sphere and an offset rod; (**h**) cross-section view with a levelling staff.

Two dedicated adapters ensure secure mounting on a broad array of surfaces and structural components. The tripod keeps the sphere stable on the ground and other flat surfaces (Figure 1). It is made of strong steel. Its mass ensures stable positioning of the openable reference sphere when measuring its centroid using diverse surveying techniques (tacheometry, levelling, GNSS) (Figure 1e,g,h). Each foot of the tripod has flat stoppers with centrally projecting tipped conic sections to ensure stable positioning in the field (Figure 1a,c–e,g,h). From the central part of the tripod (the hub) protrudes a vertical, conical spindle mating with the centring opening in the reference sphere. The adapter can be completely disassembled and is modular. Therefore, it can be adjusted to stabilise the spheres on smooth and slippery surfaces. The sharp tips attached to their respective feet with screws are then replaced with suction cups (Figure 1b,f). The tripod’s design allows for convenient and quick adaptation to the type of surface.

**Figure 2:**
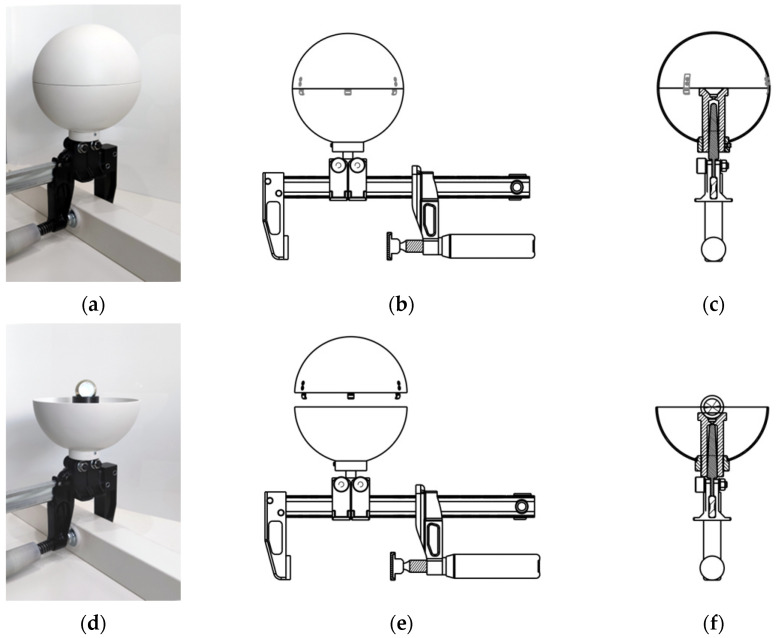
F-clamp adapter with an openable reference sphere: (**a**) perspective with a closed sphere; (**b**) side view with a closed sphere; (**c**) cross-section view with a closed sphere; (**d**) perspective with an open sphere and a retro-reflector; (**e**) side view with an open sphere; (**f**) cross-section view with a retro-reflector.

The adjustable F-clamp adapter provides stable mounting on various structural elements (balustrades, railings, beams, and brackets) of any size and material, while protecting the mounting surface (Figure 2). It is made of duralumin, a durable, low-mass material. It ensures sphere stability when its centroid is being precision-measured (Figure 2d,f). The adapter frame is a bar with grooves on both sides. It has a stopper on one end and a fixed jaw perpendicular to the bar on the other. There is a carriage with a vertical, conical spindle for the reference sphere immediately next to the fixed jaw. The bar also has a movable jaw on an arm with a threaded hole on the end. A horizontal clamping screw runs through the opening. The screw has a protective cap on one end and an ergonomic handle on the other, allowing convenient adjustment of the clamping force. The adapter’s design allows for convenient and rapid adaptation to the size of the structural element on which the sphere is mounted.

The presented spheres (Figure 1 and Figure 2) have a high sphericity, a precisely defined diameter, and good retroreflective performance, similar to other reference systems. All these characteristics improve automated detection in TLS point clouds. The novelty of the proposal lies in the fact that the coordinates of the reference sphere’s centroid can be measured directly, enabling unambiguous, metrologically reliable georeferencing of TLS data. The openable steel sphere with the adjustable cylindrical centring pin is fully compatible with the high-precision spherical prism (Figure 1e and Figure 2d,f). The adjustable pin height allows the centre to serve as a measurement point in tacheometry (Figure 1e,g, Figure 2d,f), levelling (Figure 1h), and GNSS surveys (Figure 1g). It is a unique link between TLS and traditional surveying methods. Combined with the dedicated adapters, the openable reference spheres make up a new, integrated reference system. It ensures optimal distribution of scanning targets, direct and precise determination of sphere centroid coordinates using surveying methods, and high-accuracy TLS point cloud registration and georeferencing.

### 2.2. Validation Procedure

The referencing system for TLS cloud registration and georeferencing was experimentally validated in the Surveying Equipment Laboratory of the Faculty of Environmental Engineering and Land Surveying at the University of Agriculture in Kraków. The purpose was to establish an unbiased metrological evaluation of the system’s performance. It covered the accuracy of reproduction of geometric parameters and the stability of any mounting elements under simulated real-life conditions of use. The research protocol was divided into two stages. The first stage was a metrological and calibration assessment. It focused on the quality of fit of the spheres in TLS point clouds, form errors, sphere-centre distance errors, and the quality of registration and georeferencing. The other stage verified the stability and repeatability of the F-clamps. The reference spheres were mounted on typical structural elements (such as beams, balustrades, and railings) made of various materials and having different cross-sections. This way, we could evaluate the impact of mounting conditions on the accuracy of sphere centroid positioning. The performance of the referencing system was evaluated with four modern terrestrial laser scanners from leading manufacturers (Z+F Imager 5010C [76], Riegl VZ-400 [77], Leica ScanStation P40 [78], and Trimble TX8 [79]), which enabled us to compare it across various measurement techniques and ensured the versatility of the conclusions.

#### 2.2.1. Measurement Equipment

The validation involved four surveying terrestrial laser scanners representing diverse technical approaches and ranging strategies: Z+F Imager 5010C, Riegl VZ-400, Leica ScanStation P40, and Trimble TX8. With this broad array of devices, we could compare how the original referencing system performs with phase-based ranging and ToF (time-of-flight) sensors across different acquisition accuracy and performance parameters. Z+F Imager 5010C employs phase shift between the emitted and received laser signal as a modulated continuous wave, which ensures high measurement accuracy over a limited scanning range [76]. Riegl VZ-400 uses ToF. It measures the distance based on the time of pulse propagation [77]. Its accuracy depends on the precision of time measurement and the pulse emission frequency, which limit the acquisition speed. Still, this technique is preferred for longer distances because of its greater range, at the expense of lower measurement speed and accuracy compared to phase-based systems. Leica ScanStation P40 implements the ToF method with direct time measurement aided by Waveform Digitising (WFD). It can analyse the entire pulse form, improve accuracy through internal calibration, and achieve higher acquisition speeds [78]. On the other hand, Trimble TX8’s ToF technique is amplified with Trimble Lightning. This technology can measure time to nanosecond precision and record up to a million points per second across the entire scanning range, significantly improving acquisition performance and precision [79]. The basic technical parameters and measurement modes are summarised in Table 1.

#### 2.2.2. Experimental Measurement Campaign

**Figure 3:**
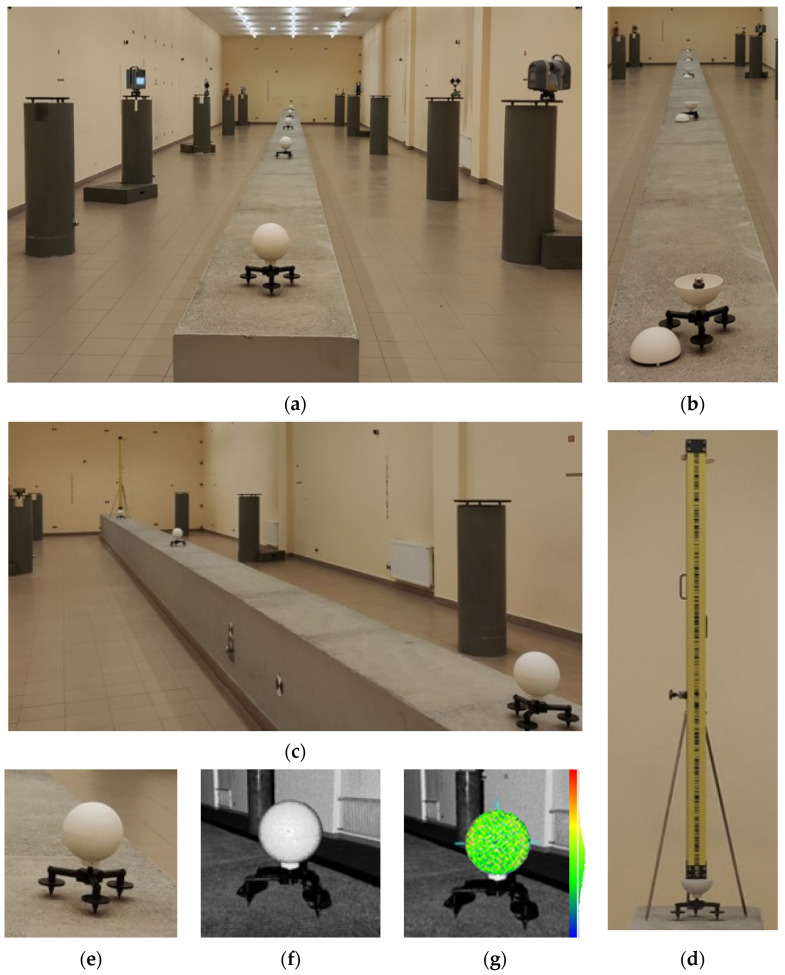
The first stage of the measurement campaign: (**a**) laser scanning of closed spheres; (**b**) tacheometric measurement of spheres’ centroids; (**c**) geodetic levelling of closed spheres; (**d**) geodetic levelling of open spheres; (**e**) an openable reference sphere on a tripod adapter; (**f**) point cloud of an openable reference sphere; (**g**) point cloud of an openable reference sphere after fitting a perfect sphere, with colour-coded residual map (range: −1.7 mm in blue to +1.5 mm in red).

The test procedures followed the VDI/VDE 2634 standard [80]. It is a common reference for evaluating the precision and accuracy of 3D scanning measurement systems. It is universally used for validating terrestrial laser scanners [70]. The standard defines a detailed method for analysing geometric errors, including form error (FE) and sphere distance error (SD). Stage I involved five original openable reference spheres distributed evenly in the test space on tripod adapters (Figure 3e). Four independent scanning sessions were performed for each scanner in different spatial configurations; the station was moved in relation to the spheres (Figure 3a). All the instruments performed scans from the same four stations within the test area to cover the surface of each sphere to the largest possible extent. The scanners were mounted on pillars with well-defined coordinates and self-centring plates. The acquisition parameters (resolution and quality mode) varied across the devices. Still, all scans followed the principles of metrological practice. Tilt compensation was applied (if available), and the data were recorded at high resolution and quality (see Table 1). We used standard, 6″ black-and-white Z+F Professional paddle targets mounted on the pillars on tribarchs and high-precision Leica GZR3 carriers that guarantee centring accuracy of 0.3 mm and plumbing accuracy of 0.5 mm/1.5 m (Figure 3a). The centroids of the spheres were measured directly with Leica TC 2003 (nominal angle measurement accuracy of 0.5″ and standard ranging error of 1 mm + 1 ppm) using a spherical prism (retroreflector) for high-accuracy measurements (Figure 3b). We also conducted geodetic levelling with Leica NA3003 and Leica GPCL2 invar bar code staffs for a reciprocal-levelling accuracy of 0.4 mm over 1 km. The levelling was performed both for closed reference spheres (Figure 3c) and for the pins with the spheres open (Figure 3d), stabilising the staff with steel props. The first stage of measurement is shown in Figure 3.

**Figure 4:**
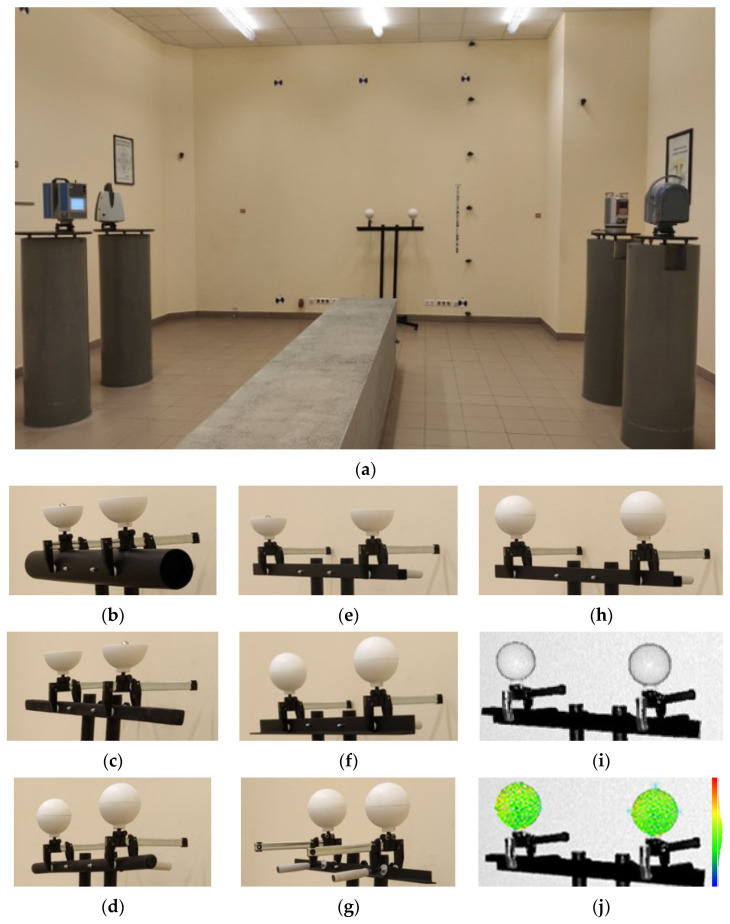
The second stage of the measurement campaign: (**a**) laser scanning of spheres on a square beam ⧠ with four scanners; (**b**) tacheometric measurement, a Ø100 mm plastic pipe; (**c**) tacheometric measurement, a square ⧠ wooden beam; (**d**) laser scanning, a round ⌾ wooden beam; (**e**) tacheometric measurement, an open ⫎ steel beam; (**f**) laser scanning, an L steel beam; (**g**) laser scanning, a T steel beam; (**h**) laser scanning, an open ⫎ steel beam; (**i**) point cloud, an open ⫎ steel beam; (**j**) point cloud after perfect sphere fitting, with colour-coded residual map (range: −2.2 mm in blue to +2.6 mm in red).

We began stage two by developing a dedicated test platform to evaluate the stability of the F-clamp adapters under conditions typical of engineering surveys. The platform’s design covered seven different mounting scenarios that reflected typical real-life cases (Figure 4a). It included construction elements of variable geometries, stiffness parameters, and materials, such as steel beams shaped like L (Figure 4e), T (Figure 4f), and ⫎ (Figure 4d,h–j), round and square wooden girders (Figure 4d,c, respectively), and a 100 mm plastic pipe (Figure 4b). This experiment involved two openable reference spheres mounted on each type of element, one by one, with the F-clamps. The closed spheres were scanned with all four laser scanners (Figure 4a). After opening them, we measured their centroids directly with Leica TC 2003 using the spherical prism (Figure 4b,c,e). The second stage of the measurement campaign is shown in Figure 4.

#### 2.2.3. Data Processing Workflow

After the data were recorded in the two stages, we proceeded to a detailed analysis of the reference spheres’ geometries in line with the VDI/VDE 2634 standard. We calculated the form error (FE) and sphere-centre distance error (SD). The procedure involved automated fitting of perfect spheres to the cloud points using the least-squares method (Least Squares Fitting) in Leica Cyclone (Cyclone CORE Version 2023.0.2). The software has sphere recognition and fitting algorithms. This approach is consistent with current research practices [66], in which commercial software serves as a reference point for testing custom sphere detection algorithms. We determined the centroid of each sphere, the diameter of a fitted perfect sphere, and fit parameters, including the root mean square error (RMSE), mean absolute error (MAE), and maximum absolute error (MaxAE). We calculated the form error (FE) using the difference between the perfect sphere radius estimated with the least-squares method (R_i_) and the actual radius of the reference sphere of 75 mm (R):

(1) $$FE=R_{i}-R$$

The sphere distance error (SD) was calculated from differences between reference sphere centroid coordinates from scanning (Δx_tls_, Δy_tls_, Δz_tls_) and reference values measured with total stations (Δx_tach_, Δy_tach_, Δz_tach_):

(2) $$SD=\sqrt{\Delta x_{tls}^{2}+\Delta y_{tls}^{2}+\Delta z_{tls}^{2}}-\sqrt{\Delta x_{tach}^{2}+\Delta y_{tach}^{2}+\Delta z_{tls}^{2}}$$

The SD values were juxtaposed with the quality threshold of four target measurement errors (TME) calculated as proposed by Fan et al. [29] as the root of the sum of squares of target measurement error for TLS (TME_TLS_) and tacheometry (TME_Tach_):

(3) $$TME=\sqrt{{\left({TME}_{TLS}\right)}^{2}+{\left({TME}_{Tach}\right)}^{2}}$$

The TME_TLS_ value, defined as a difference between the measured and actual target coordinates in the scanner’s coordinate system, is assumed at the level of RMSE of sphere fit determined for each scanner [29]. TME_Tach_ was estimated from the mean error determined from the adjustment of tacheometric measurements. Assuming double values for each sphere and then adding the values up for a pair of spheres reflects uncertainty propagation in accordance with the principle of the sum of squared errors. It provides a reasonable threshold for assessing the conformity of the reference system with accuracy requirements (±4·TME). In this case, the distance between the centroids of two spheres is a derivative quantity, determined based on two independent estimations of the centres. According to the uncertainty propagation principle, the standard overall uncertainty of the distance is the root of the sum of squares of individual uncertainties (TME) assigned to each sphere, equivalent to 2 TME for one-sided distance uncertainty. The acceptance threshold was set at twice the value, i.e., 4 TME, because the comparison involves a couple of spheres, and the threshold is two-sided. The threshold of ±4·TME serves as a formally justified criterion for evaluating the results against reference values, minimising the risk of erroneous interpretation of deviations and ensuring consistent metrological evaluation of the system.

We analysed the quality of registration and georeferencing for the reference system in the first stage of the experiment. Each scanner collected point clouds from four separate stations. The data were co-registered and transformed into a common reference frame using five openable reference spheres placed in the survey area on tripod adapters. The quality of the registration and georeferencing was assessed using a multivariate approach that included indicators typically used to validate point cloud fitting processes [81]: mean absolute error (MAE) and root mean square error (RMSE). The analyses involved both parameters reported in Leica Cyclone and using an independently calculated Post-registration Target Difference (PTD) metric proposed by Fan et al. [29] to ensure comprehensive quantitative characteristics of the orientation process, while keeping the results comparable to literature data. Post-registration Target Difference was defined as the residual difference between the coordinates of the registered and georeferenced sphere’s centroid (tie point) ($x_{tls}^{ref}$, $y_{tls}^{ref}$, $z_{tls}^{ref}$) and those measured with tacheometry (x_tach_, y_tach_, z_tach_):

(4) $$PTD=\sqrt{{\left(x_{tls}^{ref}-x_{tach}\right)}^{2}+{\left(y_{tls}^{ref}-y_{tach}\right)}^{2}+{\left(z_{tls}^{ref}-z_{tach}\right)}^{2}}$$

Values of PTD for all reference spheres were juxtaposed to estimate target registration error (TRE) in two variants as interpreted by Fan et al. [29]: TRE_MAE_ as a mean absolute error and TRE_RMS_ as the root mean square of PTD. The relationships between TRE_MAE_, TRE_RMS_, and PTD are defined in Equations (5) and (6).

(5) $${TRE}_{MAE}=\frac{1}{n}\sum_{j=1}^{n}\left|{PTD}_{i}\right|$$

(6) $${TRE}_{RMS}=\sqrt{\frac{1}{n}\sum_{j=1}^{n}{\left({PTD}_{i}\right)}^{2}}$$

Additionally, we used independent control points excluded from the transformation procedure and serving as an objective quality verifier, as recommended by Uggla and Horemuz [45]. These points were black-and-white targets in the survey area on the pillars, whose coordinates were measured with a total station in the target coordinate system down to tenths of a millimetre. The verification involved comparing the coordinates of the four control points with those of their counterparts identified in the georeferenced point cloud after transformation using the original referencing system. The deviations were compared with a threshold equal to twice the mean error in the calculation of coordinate differences due to tacheometric and TLS measurement errors. Moreover, we analysed the results of geodetic levelling for closed sphere centroids and for pins inside open spheres by juxtaposing them with theoretical values calculated from nominal sphere radii to identify any systematic errors affecting the vertical component. The purpose was to detect potential geometric deviations or mounting errors that could affect the accuracy of transformation and the end result of georeferencing.

## 3. Results

The laboratory tests validated the performance of the referencing system consisting of openable reference spheres whose centroids can be precisely measured with surveying equipment and special adapters: tripods and F-clamps, to ensure secure mounting of the spheres under diverse site conditions. The verification aimed to evaluate the system’s ability to provide high-quality TLS data registration and georeferencing under controlled experimental conditions. Considering the multidimensionality of the data and to ensure a coherent and consistent presentation, the key results are grouped into focus sections for the sphere fitting quality and form errors, sphere distance errors, registration and georeferencing quality parameters, and adapter stability evaluation.

### 3.1. Quality of Sphere Fitting and Form Errors

The procedure for evaluating the geometry of the openable reference spheres involved automated fitting of perfect spheres to the cloud points using the least squares method (Least Squares Fitting) in Leica Cyclone. The software has sphere recognition and fitting algorithms. The analysis involved 156 spheres (100 on a tripod—stage I—and 56 on an F-clamp in stage II) positioned 3–35 m from the scanners. For stage I, the spheres were fitted for individual scans and point clouds after registering four scans from a single scanner with black-and-white targets (with registration MAE below 1 mm). Table 2 summarises sphere fit parameters, i.e., the root mean square error (RMSE), mean absolute error (MAE), and maximum absolute error (MaxAE). The table additionally includes means, standard deviations, and ranges of form errors (FE) defined as the difference between the radius of a fitted perfect sphere and the actual reference sphere radius of 75 mm.

Our analysis of the quality of fit of perfect spheres demonstrated that the RMSE ranged from 0.18 to 0.85 mm across all scanners, which confirms the high consistency of spheres fitted using the least squares method with the actual distribution of points in the cloud. Values of the MAE characterise the average deviation of points from the fitted surface in any direction. They ranged from 0.13 to 0.67 mm. MaxAE specifies the maximum deviation of a single point from a fitted sphere. It was within 7 mm for all measurement configurations. The lowest RMSE, MAE, and MaxAE were found for Z+F Imager 5010C, which confirms the better stability of phase-based ranging over short and medium distances. Riegl VZ-400, with its ToF technology, had relatively higher values of all the indicators, especially over 20 m. This may be because of its noise resilience characteristics and the limited precision of ToF estimation. Despite the differences, all RMSE values were below the threshold (TME_TLS_ ≤ 1 mm), which confirms the proper accuracy of the target acquisition with TLS for engineering applications and compatibility of the openable reference spheres with the tested laser scanning systems.

The form error analysis for sphericity in accordance with VDI/VDE 2634 involved comparing the radius of a least-squares fitted sphere with the actual radius of the reference sphere equal to 75 mm. The mean FE ranged from –0.03 to 0.16 mm across the scanning systems. The smallest standard deviation of form error was identified for Z+F Imager 5010C. For Leica ScanStation P40 and Trimble TX8, FEs were slightly higher, but the largest spread was found for Riegl VZ-400. The FE distribution was normal (Shapiro–Wilk test, *p* = 0.12 > 0.05), which confirms there were no systematic errors caused by the split design of the spheres. The impact of range and incidence angle was minute. Still, FE standard deviations increased only slightly (by about 15%) for distances over 20 m. The incidence angle had no significant impact within the test range. The FE statistical parameters indicate that Z+F Imager 5010C provided a better representation than Trimble TX8, Leica ScanStation P40, and Riegl VZ-400, confirming the advantage of phase-based systems over short- and medium-range scanning. Riegl VZ-400 had the largest form errors, which correlates with its technical specifications (declared ranging accuracy of 3 mm + 10 ppm compared to 1 mm for the other systems). Still, none of the scanners exceeded 1 mm FE, and the small differences among them are negligible in the case of engineering applications.

### 3.2. Sphere Distance Error Analysis

We calculated the sphere distance error (SD) to quantify the accuracy of the representation of distances between centres of reference spheres. The error is defined as the difference between the coordinates of sphere centres from TLS point clouds and the reference values measured with a total station. As per the VDI/VDE 2634 guidelines, the analysis involved 200 distances between spheres (stage I), ranging from 5 to 30 m. The distances from stage II were excluded to avoid introducing the potential issue of adapter instability. The evaluation concerned distances between spheres fitted to individual scans and distances between spheres fitted to data from four scans registered for each scanner using black-and-white targets (whose MAE was below 1 mm). Table 3 summarises means, standard deviations, MAEs, and SD ranges for the four TLS scanners. The results were juxtaposed with fourfold target measurement errors (±4·TME) calculated as the root of the sum of squares of target measurement errors for TLS (TME_TLS_) and tacheometry (TME_Tach_). TME_TLS_ is assumed to be the average RMSE for sphere fit for individual scanners, while TME_Tach_ is derived from the mean error of tacheometry post-processing (m_p_ = 0.4 mm).

The results show that the mean SD errors ranged from –0.25 to 0.17 mm, depending on the scanning system. The SD distribution was normal (Shapiro–Wilk test, *p* = 0.15 > 0.05), which confirms there were no systematic errors from the proposed original referencing system. Similarly to FE, this analysis demonstrated that the lowest SD error and its standard deviations were identified for Z+F Imager 5010C, which uses phase-based ranging. The SD values were slightly higher for Leica ScanStation P40 and Trimble TX8, but Riegl VZ-400 had the largest spread of deviations. Also, its standard deviation was about twice that of Z+F Imager 5010C and Leica ScanStation P40. These differences are consistent with the ranging accuracies declared by these manufacturers (Table 1): Riegl VZ-400 has an accuracy of 3 mm + 10 ppm, while the others reach about 1 mm. The acceptance threshold for the validation is ±4·TME (twice the measurement accuracy for each sphere in the pair for which the distance was measured). It was defined in line with uncertainty propagation (TME_TLS_ and TME_Tach_), following the principle of the sum of squared errors. The overall distance uncertainty for two independent measurements of centres of spheres is the root of the sum of squares of their individual errors. The ±4·TME threshold has never been violated. This indicates that the SD errors are within the ranges of expected uncertainties of the referencing system caused by the accuracy of TLS and tacheometric measurements of spheres’ centres. Only a few SD values for Trimble TX8 approached the limit, but they stayed below it. These results confirm compliance with the expected accuracy declared by the manufacturers and the absence of additional systematic errors from the original referencing system, rendering it fit for high-precision engineering projects.

### 3.3. Registration and Georeferencing Quality

The accuracy and repeatability of registration and georeferencing using the proposed referencing system were evaluated using the data from stage I, as reported by commercial software Leica Cyclone, and an in-depth analysis of Post-registration Target Difference (PTD). We defined PTD as the difference between the coordinates of the reference sphere centres in an oriented TLS point cloud and those measured with a total station. The analysis included a total of 80 PTD observations (20 per scanner). Five reference spheres were identified in each scan. The PTD values were used to calculate the registration error and georeferencing errors: TRE_RMS_ (root mean square of the differences) and TRE_MAE_ (mean absolute value of the differences). Table 4 summarises the errors reported by Leica Cyclone (mean absolute error, MAE and root mean square of errors, RMS_E_), PTD statistics (standard deviation and maximum), and calculated TRE values.

Statistics for PTD demonstrated that its standard deviations ranged from 0.57 to 0.78 mm, while the maximums were from 1.41 to 2.45 mm. Z+F Imager 5010C yielded the lowest values, and Riegl VZ-400, the highest. The registration and georeferencing metrics calculated from PTD: TRE_RMS_ and TRE_MAE_ ranged from 1.05 mm to 1.57 mm and from 0.87 mm to 1.38 mm, respectively, demonstrating a similar trend. The lowest values were calculated for the phase-based Z+F Imager 5010C. Leica ScanStation P40 reached similar values. Trimble TX8 fared slightly worse, and Riegl VZ-400 (ToF-based) came in last. According to the reports in Leica Cyclone, MAE for registration and georeferencing was 1 mm for all four scanners. RMSE values based on the reported errors ranged from 1.02 to 1.66 mm, exhibiting a similar tendency: the lowest values for Z+F Imager 5010C and the highest for Riegl VZ-400. These differences are consistent with FE and SE analyses as well as the declared ranging accuracies (Table 1): 3 mm + 10 ppm for Riegl VZ-400 and around 1 mm for the other scanners.

We confirmed the reliability of the results using independent control points: four targets on pillars whose coordinates were determined with a total station down to 0.3 mm point positioning accuracy. The verification involved comparing the coordinates of the control points with those of their counterparts identified in the georeferenced point cloud after transformation using the original referencing system. The average coordinate differences varied from –0.55 mm to 0.72 mm and did not exhibit systematic deviations. Every discrepancy was below the doubled mean error of coordinate measurement, defined as a resultant of TLS measurement error, registration and georeferencing error, and tacheometry error. We then compared the results of geodetic levelling of centroids of closed spheres and their pins when open to the expected values calculated using the nominal sphere radius (75 mm). This excluded potential deviation from plumb due to mounting errors or imperfect geometry of the spheres. The average differences between the measured and expected values were 0.22 mm for closed spheres and 0.31 mm for the pins with spheres open. The maximum difference did not exceed 0.53 mm, and the residual distribution was normal, which indicates random errors. Additionally, an analysis of differences between values of the two states (closed and open) revealed an average difference of 0.21 mm, which is within the accuracy limit for geodetic levelling (0.4 mm/1 km). The absence of significant systematic deviations confirms that the designs of the reference spheres and the tripod adapters ensure sphere stability and do not introduce additional errors for the vertical component. The height representation conforms to the nominal assumptions.

### 3.4. Stability of Adjustable F-Clamp Adapters

The second stage of the experiment verified the stability of the reference sphere positioning provided by the original F-clamp adapters. The test was conducted using a dedicated platform. The seven clamping scenarios reflected typical site and structural conditions. The reference spheres were mounted on elements of various geometries and materials (such as a plastic pipe, steel beams of cross-sections resembling ⫎, ⧠, ⎿, and ⟙ as well as round and square wooden beams) using the adjustable F-clamps. Two closed spheres were scanned with all four laser scanners in each configuration. After opening them, we measured their centroids directly with a total station. Twenty-eight configurations were analysed in total (seven scenarios, four scanners). We calculated the SD for each configuration. Sphere distance error is defined as the difference between distances between sphere centres measured with TLS and the reference values measured with the total station. We also calculated the intraclass correlation coefficient (ICC), which measures the repeatability of sphere position in multiple measurements for each scenario. Table 5 shows SD error statistics (means, standard deviations, MAE, ranges), the percentage of observations out of range of ±4·TME, and ICC.

Sphere distance error statistics show that the values were within –2.11 to 1.60 mm, which is comparable to the values from stage I. The smallest standard deviation values were identified for mounting on stiff steel and wooden elements exhibiting regular geometries. The results were more scattered for smoother and more elastic elements, like a plastic pipe, or geometrically complex objects (like the open ⫎ steel beam). The results were compared with the fourfold target measurement error (±4·TME) calculated as in stage I. The upper acceptance limit of ±4·TME (aggregate uncertainty from TLS and tacheometry) was not breached. Only a few SD values for Trimble TX8 (the Ø100 mm plastic pipe) and Leica ScanStation P40 (the square wooden beam) approached the threshold. The ICC ranged from 0.90 to 0.99, which means ‘perfect’ repeatability, according to [82]. The highest values (ICC > 0.96) were obtained for spheres on steel and wooden elements of regular geometry. Still, even under the most difficult conditions involving smooth and elastic objects (the Ø100 mm plastic pipe) or complex elements (the open ⫎ steel beam), the values were relatively high (0.90–0.95). The deviations were below the acceptance threshold (±4·TME) even in these cases, which confirms the high stability of the clamp adapters. These results unambiguously indicate that the proposed F-clamp adapters guarantee high repeatability and stability of reference sphere positioning, regardless of the type of structure they are mounted on. The differences across the scenarios are minuscule and do not affect the quality of point cloud registration and georeferencing.

## 4. Discussion

### 4.1. Precision, Stability, and Reliability of the Reference System in Laboratory Measurements

The laboratory tests have unambiguously confirmed the high quality, repeatability, and stability of the original referencing system for TLS data registration and georeferencing, consisting of openable reference spheres and tripod and F-clamp adapters. The analysis of parameters of the least squares fit of perfect spheres to the point clouds demonstrated RMS values below 1 mm for all scanners. This proves very good conformity of the fitted perfect sphere to the actual distribution of points and absence of significant distortions caused by the design of the spheres. Results of the analysis of form errors (FE) calculated according to VDI/VDE 2634 have confirmed the high conformity of the sphere radius determined by the least squares method with the nominal radius of 75 mm. No FE value exceeded 1 mm. The error distribution was normal, indicating the absence of additional systematic errors from the proposed system. Similar results were obtained for sphere distance errors (SD) calculated as differences between the distances between sphere centres from TLS clouds and those measured with a total station. No result breached the acceptance threshold (±4·TME) based on TLS and tacheometry measurement error propagation. The differences were normally distributed, confirming the absence of systematic effects and making the system suitable for high-precision applications. Its high stability has also been confirmed in analyses of registration and georeferencing quality using PTD, TRE_RMS_, and TRE_MAE_. Sub-millimetre values of the parameters and results for the independent control points confirmed the absence of systematic errors and conformity of registration and georeferencing quality with the metrological assumptions. The additional verification with geodetic levelling of closed spheres and pins inside open spheres has demonstrated no vertical deviations over the nominal accuracy, confirming the vertical stability of the design of the spheres and adapters. The overall results demonstrate beyond any doubt that the original referencing system ensures reliable, stable, and repeatable registration and georeferencing of TLS point clouds, making it suitable for high-precision engineering projects and measurements requiring millimetre-level accuracy.

Our results are consistent with literature reports. Mukupa et al. [3] indicate that indirect target-based georeferencing is capable of reaching sub-millimetre accuracy and is one of the most accurate georeferencing methods. Lichti et al. [50] report similar levels of uncertainty and performance for well-designed reference targets in laboratory tests. Additionally, Kersten and Lindstaedt [70] achieved similar accuracies in distance measurement error tests using reference spheres on a 20-metre comparator track, demonstrating distance deviations below 1 mm compared to precision tacheometry. Furthermore, our research demonstrated no impact of the incidence angle on the estimation of reference sphere parameters, which is consistent with [48,54,62,63]. These authors point out the advantage of reference spheres over targets whose performance is limited by the incidence angle. The cardinal contribution of the proposed referencing system is that it addresses the primary flaw of traditional reference spheres, which is their limited use for georeferencing, as emphasised by Fryskowska [71]. Since the spheres can be opened and their centres measured directly with surveying equipment, they can be integrated into the reference frame, ensuring accurate registration and georeferencing comparable to that of commercial targets. Therefore, the system can be considered useful for high-precision engineering applications such as deformation monitoring or regular deformation and displacement analysis. Its additional benefit is that no targets need to be rotated manually to face the scanner, which can add 15% to the scanning time on average and generate positioning errors, according to Becerik-Gerber et al. [41].

The comparative analysis demonstrated that the proposed openable reference spheres are compatible with the scanners by leading brands used in the experiment (Z+F Imager 5010C, Riegl VZ-400, Leica ScanStation P40, and Trimble TX8), and any differences in FE, SD, and TRE across the models can be attributed mainly to the rangefinder design and measurement technology. The phase-based Z+F Imager 5010C achieved the lowest (best) values of the quality parameters. This could be accounted for by the characteristics of the ToF method. The analysis of the phase shift in a modulated signal results in lower range-finding noise and a tighter point distribution of the sphere, which streamlines precise geometric fit on short and medium distances. ToF-based systems, represented by Riegl VZ-400, Trimble TX8, and Leica ScanStation P40 in this case, exhibit a different structure of errors and a longer range. Riegl VZ-400, a ToF-based scanner with a declared ranging accuracy of 3 mm + 10 ppm, had higher parameters (still within the quality acceptance range). It can be due to its characteristics, data acquisition techniques, and signal processing algorithms. Leica ScanStation P40 achieved middling or positive results thanks to waveform digitisation, confirming that digital pulse analysis improves the precision of time-of-flight measurement. Trimble TX8, a scanner with Lightning technology capable of generating very high point rates, improved the statistical coverage of the sphere. This affected indirect results: high point density stabilised estimation, but it did not eliminate the effects of the rangefinder’s design. The observed differences across the laser scanners correlate with their technical specifications and findings reported in the literature. Kersten and Lindstaedt [70] described better outcomes of using the phase-based Z+F than ToF systems. Likewise, Lichti et al. [50] demonstrated a better performance of Z+F Imager 5010C compared to ToF scanners: Leica ScanStation P40 and Riegl VZ-400. Suchocki [83] emphasised that the higher precision of phase-based systems emerges from the measurement method. Therefore, our results are consistent with the literature. They also confirm that although differences between scanners matter in interpreting results and choosing the right equipment for the task, they do not disqualify the proposed referencing system, which remains compatible with devices from leading global manufacturers and ensures the accuracy required for precision surveys.

### 4.2. Prototype Evolution Supported by Initial Field Experiments

**Figure 5:**
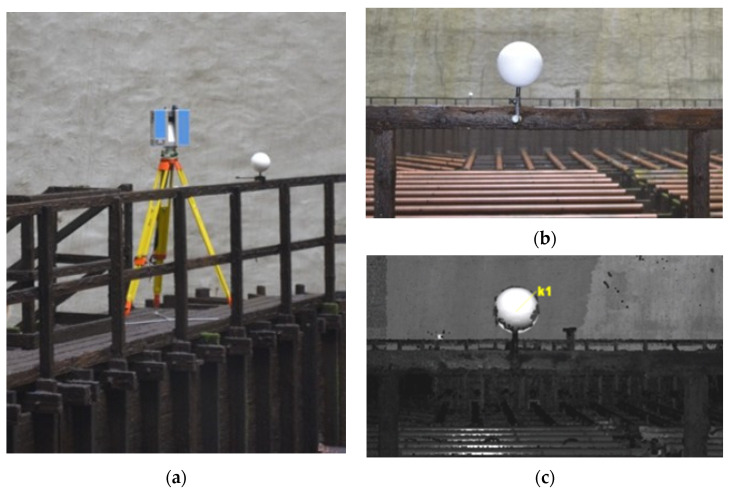
The initial versions of the adjustable F-clamp adapter during a survey inside a cooling tower: (**a**) laser scanning; (**b**) F-clamp adapter with a non-openable sphere; (**c**) cloud point with a fitted perfect sphere.

In the second stage of the experiment, which involved the dedicated platform, we evaluated the stability of the F-clamp adapters for mounting the reference spheres on structural elements of various geometries, cross-sections, and materials while protecting their surfaces. The sphere distance (SD) error analysis has demonstrated that changes in sphere centre positions relative to the reference positions did not exceed the acceptance threshold of ±4·TME. This proves that the adapters ensure stability of the openable spheres during measurements and do not introduce additional significant errors. Note that the initial designs of the F-clamp adapters were dedicated to TLS of the interior of a cooling tower [28,84], where the conditions (limited stability of wooden walkways and high humidity) commanded reference markers that did not have to be rotated. Steel spheres of 150 mm were used at the time (non-openable). They were mounted on the walkway railing with original adapters, adjustable F-clamps (Figure 5), which guaranteed stable positioning during the measurements. Thanks to the adapters, the spheres could be laid out evenly in the scanner’s field of view, ensuring the optimal TGD and stable positions, which are critical for point cloud registration. This initial design was then improved to allow for determining the coordinates of the centres of the spheres in surveys to be used for georeferencing. The experiments reported in the article confirm the successful evaluation of the adjustable F-clamp adapter and the effective resolution of the problem of measuring the centre of the sphere with surveying techniques.

**Figure 6:**
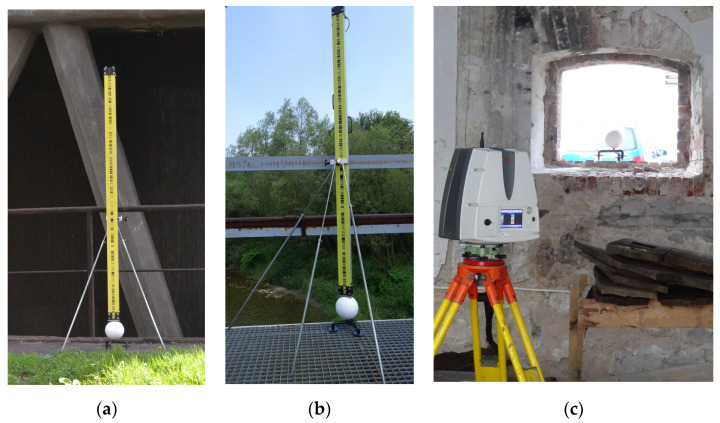
Initial versions of the tripod adapters during laser scanning of: (**a**) a cooling tower; (**b**) a steel bridge; (**c**) a heritage granary under redevelopment.

The tripod adapters followed a similar evolutionary path. Their first design was dedicated to monitoring the exterior of a cooling tower shell [28,84,85]. It was critical to ensure control over and reliability of the vertical quantities of TLS data. It was done by placing steel (unopenable) reference spheres on tripod adapters evenly over the site for geodetic levelling (Figure 6a). Adjustments of the heights of the reference spheres after controlling for their radius allowed the authors to determine the heights of the spheres’ centroids down to 0.3 mm, which is consistent with the present results for the openable spheres. This way, the spheres could serve as tie points for adjacent point clouds, improving the geometric layout of targets and enhancing the hybrid procedure for TLS data registration and georeferencing. The initial tripod adapter designs were also used in other projects. These also served as important steps in prototype evaluation. One particularly important example of how the adapters were used is a railway bridge stability survey [20,21]. Reference spheres were positioned along the bridge on tripod adapters and levelled with high precision (Figure 6b) to integrate TLS data with geodetic levelling results. This significantly improved the vertical accuracy of the point clouds, which proved critical for longitudinal surveys and dynamic tests of the steel structure under loads from rolling stock exceeding 120 tonnes. Another case study, which contributed to the evaluation to a significant degree, was monitoring surveys during a redevelopment of a historical granary in Mydlniki, Kraków, reported in [86]. The surveys of this object, using the initial version of adapters and unopenable spheres (Figure 6c), confirmed the system’s advantages, such as high stability and ergonomics (no need to use stands and space savings during transport of equipment), and versatility as the spheres can be placed on various surfaces, while retaining the optimal TGD. These experiences contributed to the evaluation and evolution of the system. The adapters were made modular so that stabilisers (fangs, suction cups) can be changed, and openable spheres were designed. The evaluation outcomes have substantially enhanced the functionality and convenience of the system, making it even more versatile and facilitating the determination of centroid coordinates directly through surveying, thereby making the spheres suitable for point cloud georeferencing. The present results unambiguously confirm the successful evaluation of the tripod adapter prototype and the effectiveness of the proposed solution concerning the precise determination of the centre of the sphere through surveying.

The field applications of the initial version of the system provided valuable insights into its behaviour in facilities with limited visibility, in structures at risk of vibration, and under volatile environmental conditions. The primary applications included scanning the interior of a cooling tower, where lighting was significantly reduced and humidity gradients were steep; monitoring a railway bridge loaded with 120 tonnes of rolling stock, with noticeable structural vibrations; and multi-epoch measurements during the redevelopment of a heritage granary in Mydlniki, Kraków, under variable weather conditions. Although the tests involved early versions of the adapters and non-openable spheres, they unambiguously confirmed positioning stability, vibration resistance, and spheres’ behaviour under volatile conditions. We find the successful application under limited visibility during the cooling tower interior scanning particularly valuable. The site tests revealed key operational advantages of the system, which are quantifiable and positively distinguish it from commercial targets on tripods. They include:

1. Substantial reduction in setup time.
  Placement of a surveying tripod, levelling, installation of a carrier, and fixing the target take one to five minutes, depending on the tripod and the operator’s experience. It takes only 10–15 s to place a reference sphere on a tripod or F-clamp adapter, meaning a single tie point can be set up 5 to 20 times faster.
2. No need to position the targets towards the scanner.
  Targets typically have to be manually set to face the scanner, which adds 15–20% to the acquisition time for four targets per station, according to our observations and literature data (Becerik-Gerber et al. [41]). The spheres do not need to be rotated or repositioned, which eliminates this stage and the risk of human error.
3. Easier transport and logistics.
  A complete set of ten reference spheres with adapters in transport configurations takes room equivalent to four tripods with carriers and targets. It means a 50% reduction in gear mass and volume, which is highly relevant to multi-station campaigns in hard-to-reach areas.

### 4.3. Limitations, Environmental Challenges, and Future Research

One of the significant limitations of the study is that it was confined to a laboratory setting. Although this helped precisely control the experimental conditions, the full complexity of the operational environment typical of actual TLS campaigns was not reflected. Despite the high level of accuracy obtained under laboratory conditions, a complete evaluation of the system’s functionality requires verification under operational conditions, where the quality of TLS data and stability of references are affected by hardly controllable factors. Field conditions involve variable natural lighting levels, structural vibrations, and temperature fluctuations, which may affect both adapter stability and properties of the sphere’s reflective surface. For instance, cycles of temperature variations may cause minor deformations of steel components, such as threaded bushes and centring pins, which could add to the centroid positioning error. Moreover, vibrations can cause momentary shifts in adapters mounted on bridge structural elements, for example. Therefore, the system’s resilience to short-term vibration and dynamic loads has to be verified. Therefore, the priority future agenda is to perform an extensive field validation of the final version of the system, including tests under complex environmental conditions: a broad range of temperatures (−10 °C to +40 °C), variable visibility, long distances typical of engineering surveying, and structural vibrations. The validation will be both static and quasi-dynamic, covering diurnal temperature and lighting cycles. Its critical part will be to evaluate the stability of adapter mounting and the long-term stability of sphere centroid coordinates. The first field experiment will involve setting up two spheres about 10 m apart and recording their relative distance every 30 min over a 24 h cycle while performing high-precision tacheometry measurements. This way, we will unambiguously evaluate the impact of environmental factors on the system’s geometry and compare the stability of distances measured with TLS and tacheometry. Further tests will concern dynamic applications. We will monitor momentary geometric shifts and microvibrations to verify the system’s performance for high-frequency monitoring of variably loaded objects or free-vibrating objects.

The next stages will involve complete calibration of the prism-sphere offset using dual-station intersection. This procedure will pinpoint the relationship between the sphere’s centroid and the prism centre to reduce systematic errors when determining the sphere centre using surveying measurements. We will simultaneously propagate the complete set of uncertainties related to the estimation of the sphere centre, including errors of prism constant and atmospheric corrections, into an aggregate standard reference uncertainty. The formal inclusion of these components in the total uncertainty budget is critical to further metrological validation of the system. It will also facilitate a complete, quantitative comparison of its properties with the existing commercial targets. Moreover, the planned tests include evaluation of the system’s compatibility with the latest-generation TLS scanners, whose accuracy, range, and acquisition rate have been improved. We will focus particularly on validating the system with at least two units of the same scanner model to determine between-unit variance and integrate it into the system’s final uncertainty budget. Moreover, it will be mandatory to record the firmware version of each scanner in future experiments, and the analysis of the influence of firmware algorithm changes on metrological results will be included in the system’s stability and repeatability assessment.

Another element of the planned field validation will be pilot tests using actual structures with diverse geometric and dynamic characteristics, such as bridges, towers, or tunnels. The tests will identify the key environmental factors (variable temperature, free vibration, lighting, and limited visibility) on the sphere-adapter arrangement and the quality of TLS point cloud registration and georeferencing. The focus will be on structural deformation monitoring, which requires longitudinal analyses, where georeferencing stability and point cloud registration quality are paramount. As demonstrated by Truong-Hong et al. [6], even the slightest discrepancies in point cloud transformations can be erroneously interpreted as actual displacement or deformation, generating undesirable measurement noise and limiting the reliability of any interpretations. The proposed referencing system can help significantly reduce such errors. The next field tests on a real-life structure will concern the measurement of a bridge section over two epochs using the final version of the system, with a parallel recording of ambient conditions (temperature, vibrations, insolation, and wind), an analysis of sphere centre coordinates stability, and evaluation of the impact of external conditions on the accuracy of registration and georeferencing. Quality control will be conducted with commercial targets measured in parallel as a reference frame to compare the performance and robustness of the two target types under actual operational conditions. Another expected direction of further research is to integrate TLS data with traditional surveying techniques. Mukupa et al. [3] emphasised that TLS often requires support in high-precision engineering projects or complements traditional methods, contributing to a more comprehensive and reliable characterisation of periodic structural changes. A synergistic approach to deformation detection that would overcome the issue of discreteness of the traditional surveying methods and expand the monitoring from selected points to the entire structure requires cohesive procedures for integrating TLS data with high-precision surveying results. The proposed referencing system can prove to be an effective tool in such processes. Future outlooks include using the system for rigorous calibration of terrestrial laser scanners, where spherical targets play an important role in evaluating and correcting instrument errors, according to Shi et al. [66].

It seems only natural to integrate the proposed referencing system with the latest sphere segmentation, detection, and fitting methods for point clouds. These methods have evolved substantially over recent years, especially in the areas of deep neural networks and local representation–based algorithms [87,88,89,90]. Future research will focus on implementing these techniques to improve the accuracy of sphere-centre positioning further and to automate TLS data registration and georeferencing. A study by Yang et al. [60] demonstrated that adaptive fitting strategies can improve sphere centre determination by at least 5.7% compared to traditional methods. Wu et al. [65] proposed a modified RANSAC method with curvature analysis. At the same time, Wang et al. [67] offered the PK-RANSAC algorithm, which improves the resilience of sphere centre extraction to noise and partial obscuration. When such innovations are integrated with the referencing system, the stability and reliability of point cloud registration and georeferencing can be substantially improved. Publications by Wang et al. [87,88], which employ mechanisms such as Taylor series-inspired local structure fitting networks and physical models of acoustic wave propagation, demonstrate that a hybrid geometric-physical representation can significantly improve segmentation performance and robustness, even with small training datasets. With such methods in the TLS data processing pipeline, the system could achieve more precise and stable sphere detection, especially in the presence of heavy noise, despite discontinuities and reduced vision. The analysis of the impact of these algorithms on the final registration quality is a paramount and priority future research direction. Future development efforts can include evaluation of the system’s potential for two-stage, coarse-to-fine registration and georeferencing. At the coarse stage, the referencing system will provide reliable initialisation data, thus reducing the risk of mismatching for methods that use natural geometric features, as they are sensitive to noise, surface discontinuities, and symmetry [30,36,37]. The fine stage will involve an ICP, GICP, FGR, or other fine registration algorithm using the initial transformation based on the reference spheres. This will improve the reliability of the entire process and help preserve the predefined reference frame, which is critical for deformation and displacement surveys or deformation monitoring. Note also that many new methods for point cloud adjustment, such as those based on keypoints [91], require transformation initialisation, which the proposed system can ensure in a clear and repeatable manner. Dedicated algorithmic procedures will be developed for the reliable detection and estimation of reference sphere centroids at the coarse stage and using the spheres and geometric features at the fine stage. The system’s integration with sophisticated machine-learning methods and shape-fitting algorithms is the central prospect for further improving the accuracy, automation, and robustness of TLS data registration. The analysis of the performance of these combinations and the assessment of their contribution to the final georeferencing quality are strategic areas of further inquiry.

**Figure 7:**
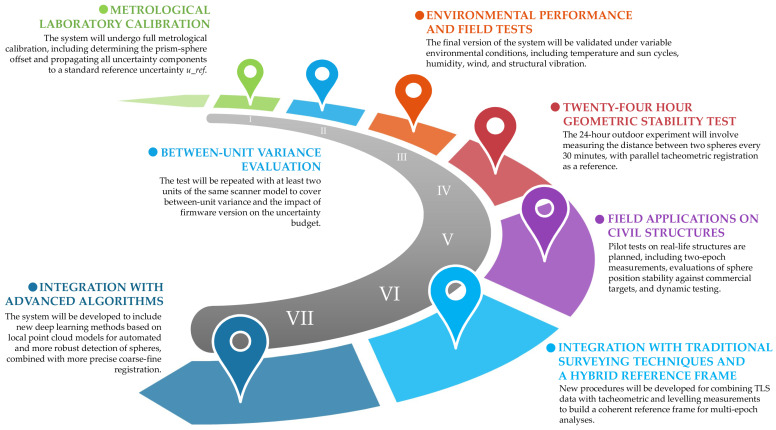
Roadmap outlining the planned operational validation and integration of the openable spherical reference target with advanced registration algorithms.

The proposed research programme is a coherent, multi-stage roadmap for the development of the referencing system (Figure 7). It covers complementary domains of metrological validation, operational field tests, and integration with sophisticated point cloud processing tools. This research path ensures a complete and systematic assessment of the system’s functionality, from analysis of geometric stability and uncertainty propagation, through environmental robustness, to the implementation of modern algorithms for improved accuracy in sphere centre positioning and the automation of TLS data registration and georeferencing. The roadmap is the essential foundation for our metrological, algorithmic, and applicational verification of the system’s usefulness for high-accuracy engineering jobs.

## 5. Conclusions

The article reports outcomes of a comprehensive validation of an original referencing system intended to improve the reliability and accuracy of terrestrial laser scanning (TLS) point cloud registration and georeferencing. The system comprises openable reference spheres, whose centroids can be directly and precisely determined using traditional surveying methods. It also includes dedicated adapters: tripods and adjustable F-clamps with which the spheres can be securely mounted on structural components of diverse geometries, facilitating the optimal distribution of the targets. The laboratory tests confirmed that the system facilitates precise stitching of point clouds acquired at various stations, transforming them to a common reference frame, and integrating with data from traditional surveying techniques (tacheometry and geodetic levelling). A verification involving four terrestrial laser scanners from leading manufacturers (Z+F Imager 5010C, Riegl VZ-400, Leica ScanStation P40, and Trimble TX8) has revealed form errors of less than 1 mm and sphere centre distance errors within tolerance limits. The results confirm that there are no significant systematic errors and that the system is fully compatible with various TLS technologies. An analysis of registration and georeferencing quality parameters has demonstrated high stability and repeatability of the system, which was additionally confirmed with independent measurements of control points. Precision levelling of the centroids of the spheres and pins inside open spheres also testifies to the sphere and adapter design’s vertical stability, eliminating the risk of mounting errors that could affect georeferencing accuracy.

By enabling direct traditional measurements of the sphere centre, the referencing system addresses the primary limitation of conventional reference spheres, facilitating the explicit integration of TLS data into the reference frame and achieving registration and georeferencing accuracies comparable to or surpassing commercial targets. It has also been demonstrated that the operational advantages, namely the reduced scanner setup time, elimination of the need to manually orient the target toward the scanner, reduced transport volume and simplified logistics, as well as the standardisation and automation of the measurement procedure, are measurable and significant, constituting a key added value of the solution. The presented empirical evidence unambiguously demonstrates that the system offers stable and repeatable results with millimetre-level accuracy, is compatible with instruments from leading brands, and has a substantial potential for further improvements, especially considering sophisticated sphere detection algorithms and coarse-to-fine registration and georeferencing. The recommended future research agenda includes verification of the system’s performance with engineering structures under real-life conditions, longitudinal verification of adapter stability under operational conditions, and analysis of the effects of various algorithms on the final metrological result. These findings will be the conclusive evaluation of the system’s value for deformation modelling and other projects requiring precise and repeatable TLS point cloud registration and georeferencing.

## 6. Patents

The presented openable reference sphere for precise registration and georeferencing of terrestrial laser scanning data is protected by a patent granted by the Patent Office of the Republic of Poland, exclusive right No. Pat. 238935 [92]. The adapters for stabilising the reference spheres on the ground and on structural elements, such as balustrades or railings, are protected utility models registered with the Patent Office of the Republic of Poland, Nos Ru.070271, Ru.070272 (initial versions), Ru.071959, and Ru.071960. The inventor is Maria Makuch.

## Figures and Tables

**Table 1:** Technical specifications of the laser scanners [76,77,78,79].

Specification	Leica ScanStation P40	Trimble TX8	Riegl VZ-400	Z+F Imager 5010C
Measurement procedure	ToF with WFD	ToF with Lightning™	ToF	Phase-based
Range [m]	max. 270	max. 340	max. 600	max. 187.3
Scan rate [points/s]	max. 1 million	max. 1 million	max. 122,000	max. 1.016 million
Field of view H/V [°]	360/270	360/317	360/100	360/320
Measurement accuracy	1.2 mm + 10 ppm	±<1 mm, 80 µrad	3 mm + 10 ppm	1 mm + 10 ppm
Measurement noise	0.4 mm @ 10 m	~0.6 mm @ 10 m	0.4 mm @ 10 m	0.3–0.5 mm @ 10 m
Measurement mode	0.002/10 m	precision 0.0067/30 m	high speed 0.020/100 m	ultra high 0.0016/10 m
Launch year	2015	2016	2009	2013

**Table 2:** Sphere fit parameters and form errors.

Scanner	Fit Quality [mm]			Form Error [mm]		
	RMSE	MAE	MaxAE	Mean FE	EstdD FE	Range FE
Z+F Imager 5010C	0.18	0.13	4	−0.03	0.20	−0.5–0.5
Riegl VZ-400	0.85	0.67	7	0.16	0.84	−1.0–1.0
Leica ScanStation P40	0.26	0.15	5	0.06	0.29	−0.5–0.5
Trimble TX8	0.36	0.28	5	−0.01	0.35	−0.5–0.5

**Table 3:** Sphere distance errors (SD) and target acquisition errors (TME_TLS_ and TME).

Scanner	SD Error [mm]				TME [mm]	
	Mean SD	EstdD SD	MAE SD	Range SD	TME_TLS_	TME
Z+F Imager 5010C	−0.25	0.78	0.70	−1.39–1.42	0.18	0.44
Riegl VZ-400	0.17	1.63	1.42	−2.66–3.11	0.85	0.94
Leica ScanStation P40	−0.20	0.84	0.66	−1.82–1.51	0.26	0.48
Trimble TX8	0.01	1.06	0.89	−1.98–2.13	0.36	0.54

**Table 4:** Registration and georeferencing quality parameters (PTD, TRE_RMS_, TRE_MAE_) and orientation errors reported in Leica Cyclone.

Scanner	Registration and Georeferencing Quality [mm]				Cyclone Error [mm]	
	EstdD PTD	Max. PTD	TRE_RMS_	TRE_MAE_	MAE	RMS_E_
Z+F Imager 5010C	0.61	1.41	1.05	0.87	1	1.02
Riegl VZ-400	0.77	2.45	1.57	1.38	1	1.66
Leica ScanStation P40	0.57	1.41	1.07	0.92	1	1.05
Trimble TX8	0.78	2.24	1.22	0.96	1	1.20

**Table 5:** F-clamp adapter stability (sphere distance error and percentage of observations above ±4·TME) and intraclass correlation coefficient.

Item	SD Error [mm]				% SD > 4·TME	ICC
	Mean SD	EstdD SD	MAE SD	Range SD		
Plastic pipe, Ø100 mm	−0.93	1.41	1.42	−2.11–1.22	0%	0.90
Steel beam ⫎	0.76	0.85	0.93	−0.47–1.60	0%	0.95
Steel beam ⧠	0.45	0.70	0.72	−0.69–0.94	0%	0.96
Steel beam ⎿	0.46	0.68	0.37	−0.72–1.13	0%	0.96
Wooden beam ⧠	−0.43	0.27	0.50	−1.87–0.14	0%	0.99
Steel beam ⟙	−0.30	0.34	0.43	−0.99–0.37	0%	0.99
Wooden beam ⌾	0.18	0.28	0.29	−0.29–0.42	0%	0.99

## Data Availability

The data used in the study are available from the corresponding author upon reasonable request.

## Abbreviations

The following abbreviations are used in this manuscript:

TLS	Terrestrial Laser Scanning
TGD	Target Geometric Distribution
ToF	Time-of-Flight
WFD	Waveform Digitizing
FE	Form Error
SD	Sphere Distance Error
TME	Target Measurement Error
TME_TLS_	TLS Target Measurement Error
TME_Tach_	Tacheometric Target Measurement Error
TRE	Transformation/Registration Error
TRE_RMS_	Root Mean Square of TRE
TRE_MAE_	Mean Absolute Error of TRE
PTD	Post-Registration Target Difference
RMSE	Root Mean Square Error
MAE	Mean Absolute Error
MaxAE	Maximum Absolute Error
ICP	Iterative Closest Point
